# Replication, falsification, and the crisis of confidence in social psychology

**DOI:** 10.3389/fpsyg.2015.00621

**Published:** 2015-05-19

**Authors:** Brian D. Earp, David Trafimow

**Affiliations:** ^1^Uehiro Centre for Practical Ethics, University of OxfordOxford, UK; ^2^Department of History and Philosophy of Science, University of CambridgeCambridge, UK; ^3^Department of Psychology, New Mexico State UniversityLas Cruces, NM, USA

**Keywords:** replication, falsification, falsifiability, crisis of confidence, social psychology, priming, philosophy of science, Karl Popper

## Abstract

The (latest) crisis in confidence in social psychology has generated much heated discussion about the importance of replication, including how it should be carried out as well as interpreted by scholars in the field. For example, what does it mean if a replication attempt “fails”—does it mean that the original results, or the theory that predicted them, have been falsified? And how should “failed” replications affect our belief in the validity of the original research? In this paper, we consider the replication debate from a historical and philosophical perspective, and provide a conceptual analysis of both replication and falsification as they pertain to this important discussion. Along the way, we highlight the importance of auxiliary assumptions (for both testing theories and attempting replications), and introduce a Bayesian framework for assessing “failed” replications in terms of how they should affect our confidence in original findings.

“Only when certain events recur in accordance with rules or regularities, as in the case of repeatable experiments, can our observations be tested—in principle—by anyone.… Only by such repetition can we convince ourselves that we are not dealing with a mere isolated ‘coincidence,’ but with events which, on account of their regularity and reproducibility, are in principle inter-subjectively testable.”

*– Karl Popper (1959, p. 45)*

## Introduction

Scientists pay lip-service to the importance of replication. It is the “coin of the scientific realm” (Loscalzo, 2012, p. 1211); “one of the central issues in any empirical science” (Schmidt, 2009, p. 90); or even the “demarcation criterion between science and nonscience” (Braude, 1979, p. 2). Similar declarations have been made about *falsifiability*, the “demarcation criterion” proposed by Popper in his seminal work of 1959 (see epigraph). As we will discuss below, the concepts are closely related—and also frequently misunderstood. Nevertheless, their regular invocation suggests a widespread if vague allegiance to Popperian ideals among contemporary scientists, working from a range of different disciplines (Jordan, 2004; Jost, 2013). The cosmologist Hermann Bondi once put it this way: “There is no more to science than its method, and there is no more to its method than what Popper has said” (quoted in Magee, 1973, p. 2).

Experimental social psychologists have fallen in line. Perhaps in part to bolster our sense of identity with the natural sciences (Danzinger, 1997), we psychologists have been especially keen to talk about replication. We want to trade in the “coin” of the realm. As Billig (2013) notes, psychologists “cling fast to the belief that the route to knowledge is through the accumulation of [replicable] experimental findings” (p. 179). The connection to Popper is often made explicit. One recent example comes from Kepes and McDaniel (2013), from the field of industrial-organizational psychology: “The lack of exact replication studies [in our field] prevents the opportunity to disconfirm research results and thus to falsify [contested] theories” (p. 257). They cite *The Logic of Scientific Discovery*.

There are problems here. First, there is the “*lack*” of replication noted in the quote from Kepes and McDaniel. If replication is so important, why isn't it being done? This question has become a source of crisis-level anxiety among psychologists in recent years, as we explore in a later section. The anxiety is due to a disconnect: between what is seen as being necessary for scientific credibility—i.e., careful replication of findings based on precisely-stated theories—and what appears to be characteristic of the field in practice (Nosek et al., 2012). Part of the problem is the lack of prestige associated with carrying out replications (Smith, 1970). To put it simply, few would want to be seen by their peers as merely “copying” another's work (e.g., Mulkay and Gilbert, 1986); and few could *afford* to be seen in this way by tenure committees or by the funding bodies that sponsor their research. Thus, while “a field that replicates its work is [seen as] rigorous and scientifically sound”—according to Makel et al. (2012)—psychologists who actually conduct those replications “are looked down on as bricklayers and not [as] advancing [scientific] knowledge” (p. 537). In consequence, actual replication attempts are rare.

A second problem is with the reliance on Popper—or, at any rate, a first-pass reading of Popper that seems to be uninformed by subsequent debates in the philosophy of science. Indeed, as critics of Popper have noted, since the 1960s and consistently thereafter, neither his notion of falsification nor his account of experimental replicability seem strictly amenable to being put into practice (e.g., Mulkay and Gilbert, 1981; see also Earp, 2011)—at least not without considerable ambiguity and confusion. What is more, they may not even be fully coherent as stand-alone “abstract” theories, as has been repeatedly noted as well (cf. Cross, 1982).

The arguments here are familiar. Let us suppose that—at the risk of being accused of laying down bricks—Researcher B sets up an experiment to try to “replicate” a controversial finding that has been reported by Researcher A. She follows the original methods section as closely as she can (assuming that this has been published in detail; or even better, she simply asks Researcher A for precise instructions). She calibrates her equipment. She prepares the samples and materials just so. And she collects and then analyzes the data. If she gets a different result from what was reported by Researcher A—what follows? Has she “falsified” the other lab's theory? Has she even shown the original *result* to be erroneous in some way?

The answer to both of these questions, as we will demonstrate in some detail below, is “no.” Perhaps Researcher B made a mistake (see Trafimow, 2014). Perhaps the other lab did. Perhaps one of B's research assistants wrote down the wrong number. Perhaps the original effect *is* a genuine effect, but can only be obtained under specific conditions—and we just don't know yet what they are (Cesario, 2014). Perhaps it relies on “tacit” (Polanyi, 1962) or “unofficial” (Westen, 1988) experimental knowledge that can only be acquired over the course of several years, and perhaps Researcher B has not yet acquired this knowledge (Collins, 1975).

Or perhaps the original effect is *not* a genuine effect, but Researcher A's theory can actually accommodate this fact. Perhaps Researcher A can abandon some auxiliary hypothesis, or take on board another, or re-formulate a previously unacknowledged background assumption—or whatever (cf. Lakatos, 1970; Cross, 1982; Folger, 1989). As Lakatos (1970) once put it: “given sufficient imagination, any theory… can be permanently saved from ‘refutation’ by some suitable adjustment in the background knowledge in which it is embedded” (p. 184). We will discuss some of these potential “adjustments” below. The upshot, however, is that we simply do not know, and cannot know, exactly what the implications of a given replication attempt are, no matter which way the data come out. There are no critical tests of theories; and there are no objectively decisive replications.

Popper (1959) was not blind to this problem. “In point of fact,” he wrote, in an under-appreciated passage of his famous book, “no *conclusive* disproof of a theory can ever be produced, for it is always possible to say that the experimental results are not reliable, or that the discrepancies which are asserted to exist between the experimental results and the theory are only apparent” (p. 50, emphasis added). Hence as Mulkay and Gilbert (1981) explain:

… *in relation to [actual] scientific practice, one can only talk of positive and negative results, and not of proof or disproof. Negative results, that is, results which seem inconsistent with a given hypothesis [or with a putative finding from a previous experiment], may incline a scientist to abandon [the] hypothesis but they will never require him to abandon it… Whether or not he does so may depend on the amount and quality of positive evidence, on his confidence in his own and others' experimental skills and on his ability to conceive of alternative interpretations of the negative findings. (p. 391)*

Drawing hard and fast conclusions, therefore, about “negative” results—such as those that may be produced by a “failed” replication attempt—is much more difficult than Kepes and McDaniel seem to imagine (see e.g., Chow, 1988 for similarly problematic arguments). This difficulty may be especially acute in the field of psychology. As Folger (1989) notes, “Popper himself believed that too many theories, *particularly in the social sciences*, were constructed so loosely that they could be stretched to fit any conceivable set of experimental results, making them… devoid of testable content” (p. 156, emphasis added). Furthermore, as Collins (1985) has argued, the less secure a field's foundational theories—and especially at the field's “frontier”—the more room there is for disagreement about what should “count” as a proper replication^1^.

Related to this problem is that it can be difficult to know *in what specific sense* a replication study should be considered to be “the same” as the original (e.g., Van IJzendoorn, 1994). Consider that the goal for these kinds of studies is to rule out flukes and other types of error. Thus, we want to be able to say that the *same experiment*, if repeated one more time, would produce the *same result* as was originally observed. But an original study and a replication study cannot, by definition, be identical—at the very least, some time will have passed and the participants will all be new^2^—and if we don't yet know which differences are theory-relevant, we won't be able to control for their effects. The problem with a field like psychology, whose theoretical predictions are often “constructed so loosely,” as noted above, is precisely that we do not know—or at least, we do not in a large number of cases—which differences are in fact relevant to the theory.

Finally, human behavior is notoriously complex. We are not like billiard balls, or beavers, or planets, or paramecia (i.e., relatively simple objects or organisms). This means that we should *expect* our behavioral responses to vary across a “wide range of moderating individual difference and experimental context variables” (Cesario, 2014, p. 41)—many of which are not yet known, and some of which may be difficult or even impossible to uncover (Meehl, 1990a). Thus, in the absence of “well-developed theories for specifying such [moderating] variables, the conclusions of replication failures will be ambiguous” (Cesario, 2014, p. 41; see also Meehl, 1978).

### Summing up the problem

Hence we have two major points to consider. First, due to a lack of adequate incentives in the reward structure of professional science (e.g., Nosek and the Open Science Collaboration, 2012), actual replication attempts are rarely carried out. Second, to the extent that they are carried out, it can be well-nigh impossible to say conclusively what they mean, whether they are “successful” (i.e., showing similar, or apparently similar, results to the original experiment) or “unsuccessful” (i.e., showing different, or apparently different, results to the original experiment). Thus, Collins (1985) came to the conclusion that, in physics at least, disputes over contested findings are likelier to be resolved by *social and reputational negotiations*—over, e.g., who should be considered a competent experimenter—than by any “objective” consideration of the experiments themselves. Meehl (1990b) drew a similar conclusion about the field of social psychology, although he identified sheer boredom (rather than social/reputational negotiation) as the alternative to decisive experimentation:

… *theories in the “soft areas” of psychology have a tendency to go through periods of initial enthusiasm leading to large amounts of empirical investigation with ambiguous over-all results. This period of infatuation is followed by various kinds of amendment and the proliferation of ad hoc hypotheses. Finally, in the long run, experimenters lose interest rather than deliberately discard a theory as clearly falsified. (p. 196)*

So how shall we take stock of what has been said? A cynical reader might conclude that—far from being a “demarcation criterion between science and nonscience”—replication is actually closer to being a waste of time. Indeed, if even replications in *physics* are sometimes not conclusive, as Collins (1975, 1981, 1985) has convincingly shown, then what hope is there for replications in psychology?

Our answer is simply as follows. Replications do not need to be “conclusive” in order to be *informative*. In this paper, we highlight some of the ways in which replication attempts can be more, rather than less, informative, and we discuss—using a Bayesian framework—how they can reasonably affect a researcher's confidence in the validity of an original finding. The same is true of “falsification.” Whilst a scientist should not simply abandon her favorite theory on account of a single (apparently) contradictory result—as Popper himself was careful to point out^3^ (1959, pp. 66–67; see also Earp, 2011)—she might reasonably be open to doubt it, given *enough* disconfirmatory evidence, and assuming that she had stated the theory precisely. Rather than being a “waste of time,” therefore, experimental replication of one's own and others' findings can be a useful tool for restoring confidence in the reliability of basic effects—provided that certain conditions are met. The work of the latter part of this essay is to describe and to justify at least a few of those essential conditions. In this context, we draw a distinction between “conceptual” or “reproductive” replications (cf. Cartwright, 1991)—which may conceivably be used to bolster confidence in a particular *theory*—and “direct” or “close” replications, which may be used to bolster confidence in a *finding* (Schmidt, 2009; see also Earp et al., 2014). Since it is doubt about the *findings* that seems to have prompted the recent “crisis” in social psychology, it is the latter that will be our focus. But first we must introduce the crisis.

## The (latest) crisis in social psychology and calls for replication

“Is there currently a crisis of confidence in psychological science reflecting an unprecedented level of doubt among practitioners about the reliability of research findings in the field? It would certainly appear that there is.” So write Pashler and Wagenmakers (2012, p. 529) in a recent issue of *Perspectives on Psychological Science*. The “crisis” is not unique to psychology; it is rippling through biomedicine and other fields as well (Ioannidis, 2005; Loscalzo, 2012; Earp and Darby, 2015)—but psychology will be the focus of this paper, if for no other reason than that the present authors have been closer to the facts on the ground.

Some of the causes of the crisis are fairly well known. In 2011, an eminent Dutch researcher confessed to making up data and experiments, producing a résumé-full of “findings” that he had simply invented out of whole cloth (Carey, 2011). He was outed by his own students, however, and not by peer review nor by any attempt to replicate his work. In other words, he might just as well have *not* been found out, had he only been a little more careful (Stroebe et al., 2012). An unsettling prospect was thus aroused: Could other fraudulent “findings” be circulating—undetected, and perhaps even undetectable—throughout the published record? After an exhaustive analysis of the Dutch fraud case, Stroebe et al. (2012) concluded that the notion of self-correction in science was actually a “myth” (p. 670); and others have offered similar pronouncements (Ioannidis, 2012a).

But fraud, it is hoped, is rare. Nevertheless, as Ioannidis (2005, 2012a) and others have argued, the line between explicitly fraudulent behavior and merely “questionable” research practices is perilously thin, and the latter are probably common. John et al. (2012) conducted a massive, anonymous survey of practicing psychologists and showed that this conjecture is likely correct. Psychologists admitted to such questionable research practices as failing to report all of the dependent measures for which they had collected data (78%)^4^, collecting additional data after checking to see whether preliminary results were statistically significant (72%), selectively reporting studies that “worked” (67%), claiming to have predicted an unexpected finding (54%), and failing to report all of the conditions that they ran (42%). Each of these practices alone, and even more so when combined, reduces the interpretability of the final reported statistics, casting doubt upon any claimed “effects” (e.g., Simmons et al., 2011).

The motivation behind these practices, though not necessarily conscious or deliberate, is also not obscure. Professional journals have long had a tendency to publish only or primarily novel, “statistically significant” effects, to the exclusion of replications—and especially “failed” replications—or other null results. This problem, known as “publication bias,” leads to a file-drawer effect whereby “negative” experimental outcomes are simply “filed away” in a researcher's bottom drawer, rather than written up and submitted for publication (e.g., Rosenthal, 1979). Meanwhile, the “questionable research practices” carry on in full force, since they increase the researcher's chances of obtaining a “statistically significant” finding—whether it turns out to be reliable or not.

To add insult to injury, in 2012, an acrimonious public skirmish broke out in the form of dueling blog posts between the distinguished author of a classic behavioral priming study^5^ and a team of researchers who had questioned his findings (Yong, 2012). The disputed results had already been cited more than 2000 times—an extremely large number for the field—and even been enshrined in introductory textbooks. What if they did turn out to be a fluke? Should other “priming studies” be double-checked as well? Coverage of the debate ensued in the mainstream media (e.g., Bartlett, 2013).

Another triggering event resulted in “widespread public mockery” (Pashler and Wagenmakers, 2012, p. 528). In contrast to the fraud case described above, which involved intentional, unblushing deception, the psychologist Daryl Bem relied on well-established and widely-followed research and reporting practices to generate an apparently fantastic result, namely evidence that participants' current responses could be influenced by future events (Bem, 2011). Since such paranormal precognition is inconsistent with widely-held theories about “the fundamental nature of time and causality” (Lebel and Peters, p. 371), few took the findings seriously. Instead, they began to wonder about the “well-established and widely-followed research and reporting practices” that had sanctioned the findings in the first place (and allowed for their publication in a leading journal). As Simmons et al. (2011) concluded—reflecting broadly on the state of the discipline—“it is unacceptably easy to publish ‘statistically significant’ evidence consistent with *any* hypothesis” (p. 1359)^6^.

The main culprit for this phenomenon is what Simmons et al. (2011) identified as *researcher degrees of freedom*:

In the course of collecting and analyzing data, researchers have many decisions to make: Should more data be collected? Should some observations be excluded? Which conditions should be combined and which ones compared? Which control variables should be considered? Should specific measures be combined or transformed or both?… It is rare, and sometimes impractical, for researchers to make all these decisions beforehand. Rather, it is common (and accepted practice) for researchers to explore various analytic alternatives, to search for a combination that yields “statistical significance” and to then report only what “worked.” (p. 1359)

One unfortunate consequence of such a strategy—involving, as it does, some of the very same questionable research practices later identified by John et al. (2012) in their survey of psychologists—is that it inflates the possibility of producing a false positive (or a Type 1 error). Since such practices are “common” and even “accepted,” the literature may be replete with erroneous results. Thus, as Ioannidis (2005) declared after performing a similar analysis in his own field of biomedicine, “*most* published research findings” may be “false” (p. 0696, emphasis added). This has led to the “unprecedented level of doubt” referred to by Pashler and Wagenmakers (2012) in the opening quote to this section.

This not the first crisis for psychology. Giner-Sorolla (2012) points out that “crises” of one sort or another “have been declared regularly at least since the time of Wilhelm Wundt”—with turmoil as recent as the 1970s inspiring particular déjà vu (p. 563). Then, as now, a string of embarrassing events—including the publication in mainstream journals of literally unbelievable findings^7^—led to “soul searching” amongst leading practitioners. Standard experimental methods, statistical strategies, reporting requirements, and norms of peer review were all put under the microscope; numerous sources of bias were carefully rooted out (e.g., Greenwald, 1975). While various calls for reform were put forward—some more energetically than others—a single corrective strategy seemed to emerge from all the din: *the need for psychologists to replicate their work*. Since “*all* flawed research practices yield findings that cannot be reproduced,” critics reasoned, replication could be used to separate the wheat from the chaff (Koole and Lakens, 2012, p. 608, emphasis added; see also Elms, 1975).

The same calls reverberate today. “For psychology to truly adhere to the principles of science,” write Ferguson and Heene (2012), “the need for replication of research results [is] important… to consider” (p. 556). LeBel and Peters (2011) put it like this: “Across all scientific disciplines, close replication is the gold standard for corroborating the discovery of an empirical phenomenon” and “the importance of this point for psychology has been noted many times” (p. 375). Indeed, “leading researchers [in psychology]” agree, according to Francis (2012), that “experimental replication is the final arbiter in determining whether effects are true or false” (p. 585).

We have already seen that such calls must be heeded with caution: replication is not straightforward, and the outcome of replication studies may be difficult to interpret. Indeed they can never be conclusive on their own. But we suggested that replications could be more or less *informative*; and in the following sections we discuss some strategies for making them “more” rather than “less.” We begin with a discussion of “direct” vs. “conceptual” replication.

## Increasing replication informativeness: “direct” vs. “conceptual” replication

In a systematic review of the literature, encompassing multiple academic disciplines, Gómez et al. (2010) identified 18 different types of replication. Three of these were from Lykken (1968), who drew a distinction between “literal,” “operational,” and “constructive”—which Schmidt (2009) then winnowed down (and re-labeled) to arrive at “direct” and “conceptual” in an influential paper. As Makel et al. (2012) have pointed out, it is Schmidt's particular framework that seems to have crystallized in the field of psychology, shaping most of the subsequent discussion on this issue. We have no particular reason to rock the boat; indeed these categories will suit our argument just fine.

The first step in making a replication *informative* is to decide what specifically it is for. “Direct” replications and “conceptual” replications are “for” different things; and assigning them their proper role and function will be necessary for resolving the crisis. First, some definitions:

A “direct” replication may be defined as an experiment that is intended to be as similar to the original as possible (Schmidt, 2009; Makel et al., 2012). This means that along *every* conceivable dimension—from the equipment and materials used, to the procedure, to the time of day, to the gender of the experimenter, etc.—the replicating scientist should strive to avoid making any kind of change or alteration. The purpose here is to “check” the original results. Some changes will be inevitable, of course; but the point is that *only* the inevitable changes (such as the passage of time between experiments) are ideally tolerated in this form of replication. In a “conceptual” replication, by contrast, at least certain elements of the original experiment are *intentionally* altered, (ideally) systematically so, toward the end of achieving a very different sort of purpose—namely to see whether a given phenomenon, assuming that it is reliable, might obtain across a range of variable conditions. But as Doyen et al. (2014) note in a recent paper:

*The problem with conceptual replication in the absence of direct replication is that there is no such thing as a “conceptual failure to replicate.” A failure to find the same “effect” using a different operationalization can be attributed to the differences in method rather than to the fragility of the original effect. Only the successful conceptual replications will be published, and the unsuccessful ones can be dismissed without challenging the underlying foundations of the claim. Consequently, conceptual replication without direct replication is unlikely to change beliefs about the underlying effect (p. 28)*.

In simplest terms, therefore, a “direct” replication seeks to validate *a particular fact or finding*; whereas a “conceptual” replication seeks to validate the *underlying theory or phenomenon*—i.e., the theory that has been proposed to “predict” the effect that was obtained by the initial experiment—as well to establish the boundary conditions within which the theory holds true (Nosek et al., 2012). The latter is impossible without the former. In other words, if we cannot be sure that our finding is reliable to begin with (because it turns out to have been a coincidence, or else a false alarm due to questionable research practices, publication bias, or fraud), then we are in no position to begin testing the theory by which it is supposedly explained (Cartwright, 1991; see also Earp et al., 2014).

Of course both types of replication are important, and there is no absolute line between them. Rather, as Asendorpf et al. (2013) point out, “direct replicability [is] one extreme pole of a continuous dimension extending to broad generalizability [via ‘conceptual’ replication] at the other pole, ranging across multiple, theoretically relevant facets of study design” (p. 139). Collins made a similar point in 1985 (e.g., p. 37). But so long as we remain largely ignorant about exactly which “facets of study design” are “theoretically relevant” to begin with—as is the case with much of current social psychology (Meehl, 1990b), and nearly all of the most heavily-contested experimental findings—we need to orient our attention more toward the “direct” end of the spectrum^8^.

How else can replication be made more *informative*? Brandt et al. (2014)'s “Replication Recipe” offers several important factors, one of which must be highlighted to begin with. This is their contention that a “convincing” replication should be carried out *outside the lab of origin*. Clearly this requirement shifts away from the “direct” extreme of the replication gradient that we have emphasized so far, but such a change from the original experiment, in this case, is justified. As Ioannidis (2012b) points out, replications by the original researchers—while certainly important and to be encouraged as a preliminary step—are not sufficient to establish “convincing” experimental reliability. This is because allegiance and confirmation biases, which may apply especially to the original team, would be less of an issue for independent replicators.

Partially against this view, Schnall (2014, np) argues that “authors of the original work should be allowed to participate in the process of having their work replicated.” On the one hand, this might have the desirable effect of ensuring that the replication attempt faithfully reproduces the original procedure. It seems reasonable to think that the original author would know more than anyone else about how the original research was conducted—so her viewpoint is likely to be helpful. On the other hand, however, too much input by the original author could compromise the independence of the replication: she might have a strong motivation to make the replication a success, which could subtly influence the results (see Earp and Darby, 2015). Whichever position one takes on the appropriate degree of input and/or oversight from the original author, however, Schnall (2014, np) is certainly right to note that “the quality standards for replications need to be at least as high as for the original findings. Competent evaluation by experts is absolutely essential, and is especially important if replication authors have no prior expertise with a given research topic.”

Other ingredients in increasing the informativeness of replication attempts include: (1) carefully defining the effects and methods that the researchers intend to replicate; (2) following as exactly as possible the methods of the original study (as described above); (3) having high statistical power (i.e., an adequate sample size to detect an effect if one is really present); (4) making complete details about the replication available, so that interested experts can fully evaluate the replication attempt (or attempt another replication themselves); and (5) evaluating the replication results, comparing them critically to the results of the study (Brandt et al., 2014, p. 218, paraphrased). This list is not exhaustive, but it gives a concrete sense of how “stabilizing” procedures (see Radder, 1992) can be employed to give greater credence to the quality and informativeness of replication efforts.

## Replication, falsification, and auxiliary assumptions

Brandt et al.'s (2014) “replication recipe” provides a vital tool for researchers seeking to conduct high quality replications. In this section, we offer an additional “ingredient” to the discussion, by highlighting the role of *auxiliary assumptions* in increasing replication informativeness, specifically as these pertain to the relationship between replication and falsification. Consider the logical fallacy of affirming the consequent that provided an important basis for Popper's falsification argument.

**Table:** 

If the theory is true,	
an observation should occur (*T* → *O*)	(Premise 1)
The observation occurs (*O*)	(Premise 2)
Therefore, the theory is true (*T*)	(Conclusion)

Obviously, the conclusion does not follow. Any number of things might have led to the observation that have nothing to do with the theory being proposed (see Earp, 2015 for a similar argument). On the other hand, denying the consequent (*modus tollens*) does invalidate the theory, strictly according to the logic given:

**Table:** 

If the theory is true,	
an observation should occur (*T* → *O*)	(Premise 1)
The observation does not occur (~ *O*)	(Premise 2)
Therefore, the theory is not true (~ *T*)	(Conclusion)

Given this logical asymmetry, then, between affirming and denying the consequent of a theoretical prediction (see Earp and Everett, 2013), Popper opted for the latter. By doing so, he famously defended a strategy of disconfirming rather than confirming theories. Yet if the goal is to disconfirm theories, then the theories must be capable of being disconfirmed in the first place; hence, a basic requirement of scientific theories (in order to count as properly scientific) is that they have this feature: they must be *falsifiable*.

As we hinted at above, however, this basic framework is an oversimplification. As Popper himself noted, and as was made particularly clear by Lakatos (1978; also see Duhem, 1954; Quine, 1980), scientists do not derive predictions only from a given theory, but rather from a combination of the theory and *auxiliary assumptions*. The auxiliary assumptions are not part of the theory proper, but they serve several important functions. One of these functions is to show the link between the sorts of outcomes that a scientist can actually observe (i.e., by running an experiment), and the non-observable, “abstract” content of the theory itself. To pick one classic example from psychology, according to the theory of reasoned action (e.g., Fishbein, 1980), attitudes determine subjective norms. One implication of this theoretical assumption is that researchers should be able to obtain strong correlations between attitudes and behavioral intentions. But this assumes, among other things, that a check mark on an attitude scale really indicates a person's attitude, and that a check mark on an intention scale really indicates a person's intention. The theory of reasoned action has nothing to say about whether check marks on scales indicate attitudes or intentions; these are assumptions that are peripheral to the basic theory. They are *auxiliary assumptions* that researchers use to connect non-observational terms such as “attitude” and “intention” to observable phenomena such as check marks. Fishbein and Ajzen (1975) recognized this and took great pains to spell out, as well as possible, the auxiliary assumptions that best aid in measuring theoretically relevant variables (see also Ajzen and Fishbein, 1980).

The existence of auxiliary assumptions complicates the project of falsification. This is because the major premise of the *modus tollens* argument—denying the consequent of the theoretical prediction—must be stated somewhat differently. It must be stated like this: “If the theory is true *and a set of auxiliary assumptions is true*, an observation should occur.” Keeping the second premise the same implies that either the theory is not true or that at least one auxiliary assumption is not true, as the following syllogism (in symbols only) illustrates.

**Table:** 

*T* & (*A*_1_ & *A*_2_ … *A_n_*) → *O*	(Premise 1)
~ O	(Premise 2)
∴ ~ *T or* ~ (*A*_1_ & *A*_2_ … *A_n_*) =	
~ *T or* ~ *A*_1_ *or* ~ *A*_2_ … ~ *A_n_*	(Conclusion)

Consider an example. It often is said that Newton's gravitational theory predicted where planets would be at particular times. But this is not precisely accurate. It would be more accurate to say that such predictions were derived from a combination of Newton's theory and auxiliary assumptions not contained in that theory (e.g., about the present locations of the planets). To return to our example about attitudes and intentions from psychology, consider the mini-crisis in social psychology from the 1960s, when it became clear to researchers that attitudes—the kingly construct—failed to predict behaviors. Much of the impetus for the theory of reasoned action (e.g., Fishbein, 1980) was Fishbein's realization that there was a problem with attitude measurement at the time: when this problem was fixed, strong attitude-behavior (or at least attitude-intention) correlations became the rule rather than the exception. This episode provides a compelling illustration of a case in which attention to the auxiliary assumptions that bore on actual measurement played a larger role in resolving a crisis in psychology than debates over the theory itself.

What is the lesson here? Due to the fact that failures to obtain a predicted observation can be blamed either on the theory itself or on at least one auxiliary assumption, absolute theory falsification is about as problematic as is absolute theory verification. In the Newton example, when some of Newton's planetary predictions were shown to be wrong, he blamed the failures on incorrect auxiliary assumptions rather than on his theory, arguing that there were additional but unknown astronomical bodies that skewed his findings—which turned out to be a correct defense of the theory. Likewise, in the attitude literature, the theoretical connection between attitudes and behaviors turned out to be correct (as far as we know) with the problem having been caused by incorrect auxiliary assumptions pertaining to attitude measurement.

There is an additional consequence to the necessity of giving explicit consideration to one's auxiliary assumptions. Suppose, as often happens in psychology, that a researcher deems a theory to be unfalsifiable because he or she does not see any testable predictions. Is the theory really unfalsifiable or is the problem that the researcher has not been sufficiently thorough in identifying the necessary auxiliary assumptions that would lead to falsifiable predictions? Given that absolute falsification is impossible, and that researchers are therefore limited to some kind of “reasonable” falsification, Trafimow (2009) has argued that many allegedly unfalsifiable theories are reasonably falsifiable after all: it is just a matter of researchers having to be more thoughtful about considering auxiliary assumptions. Trafimow documented examples of theories that had been described as unfalsifiable that one could in fact falsify by proposing better auxiliary assumptions than had been imagined by previous researchers.

The notion that auxiliary assumptions can vary in quality is relevant for replication. Consider, for example, the case alluded to earlier regarding a purported failure to replicate Bargh et al.'s (1996) famous priming results. In the replication attempt of this well-known “walking time” study (Doyen et al., 2012), laser beams were used to measure the speed with which participants left the laboratory, rather than students with stopwatches. Undoubtedly, this adjustment was made on the basis of a reasonable auxiliary assumption that methods of measuring time that are less susceptible to human idiosyncrasies would be superior to methods that are more susceptible to them. Does the fact that the failed replication was not exactly like the original experiment disqualify it as invalid? At least with regard to this particular feature of this particular replication attempt, the answer is clearly “no.” If a researcher uses a better auxiliary assumption than in the original experiment, this should add to its validity rather than subtract from it^9^.

But suppose, for a particular experiment, that we are not in a good position to judge the superiority of alternative auxiliary assumptions. We might invoke what Meehl (1990b) termed the *ceteris paribus* (all else equal) assumption. This idea, applied to the issue of direct replications, suggests that for researchers to be confident that a replication attempt is a valid one, the auxiliary assumptions in the replication have to be sufficiently similar to those in the original experiment that any differences in findings cannot reasonably be attributed to differences in the assumptions. Put another way, all of the unconsidered auxiliary assumptions should be indistinguishable in the relevant way: that is, all have to be sufficiently equal or sufficiently right or sufficiently irrelevant so as not to matter to the final result.

What makes it allowable for a researcher to make the *ceteris paribus* assumption? In a strict philosophical sense, of course, it is not allowable. To see this, suppose that Researcher A has published an experiment, Researcher B has replicated it, but the replication failed. If Researcher A claims that Researcher B made a mistake in performing the replication, or just got unlucky, there is no way to disprove Researcher A's argument absolutely. But suppose that Researchers C, D, E, and F also attempt replications, and also fail. It becomes increasingly difficult to support the contention that Researchers B–F all “did it wrong” or were unlucky, and that we should continue to accept Researcher A's version of the experiment. Even if a million researchers attempted replications, and all of them failed, it is theoretically possible that Researcher A's version is the unflawed one and all the others are flawed. But most researchers would conclude (and in our view, would be right to conclude) that it is more likely that it is Researcher A who got it wrong and not the million researchers who failed to replicate the observation. Thus, we are not arguing that replications, whether successful or not, are definitive. Rather, our argument is that replications (of sufficient quality) are informative.

## Introducing a bayesian framework

To see why this is the case, we shall employ a Bayesian framework similar to Trafimow (2010). Suppose that an aficionado of Researcher A believes that the prior probability of anything Researcher A said or did is very high. Researcher B attempts a replication of an experiment by Researcher A and fails. The aficionado might continue confidently to believe in Researcher A's version, but the aficionado's confidence likely would be decreased slightly. Well then, as there are more replication failures, the aficionado's confidence would continue to decrease accordingly, and at some point the decrease in confidence would push the aficionado's confidence below the 50% mark, in which case the aficionado would put more credence in the replication failures than on the success obtained by Researcher A.

In the foregoing scenario, we would want to know the probability that the original result is actually true given Researcher B's replication failure [*p* (*T*|*F*)]. As Equation (1) shows, this depends on the aficionado's prior level of confidence that the original result is true [*p* (*T*)], the probability of failing to replicate given that the original result is true [*p* (*F*|*T*)], and the probability of failing to replicate [*p* (*F*)], as Equation (1) shows.

(1) $$p\left(T|F\right)=\frac{p\left(T\right)p\left(F|T\right)}{p\left(F\right)}$$

Alternatively, we could frame what we want to know in terms of a confidence ratio that the original result is true or not true given the failure to replicate $\left[\frac{p(T|F)}{p(\sim T|F)}\right]$. This would be a function of the aficionado's prior confidence ratio about the truth of the finding $\left[\frac{p\left(T\right)}{p\left(\sim T\right)}\right]$ and the ratio of probabilities of failing given that the original result is true or not $\left[\frac{p\left(F|T\right)}{p\left(F|\sim T\right)}\right]$. Thus, Equation (2) gives the posterior confidence ratio.

(2) $$\frac{p(T|F)}{p(\sim T|F)}=\frac{p\left(T\right)}{p\left(\sim T\right)}\frac{p\left(F|T\right)}{p\left(F|\sim T\right)}$$

Suppose that the aficionado is a very strong one, so that the prior confidence ratio is 50. In addition, the probability ratio pertaining to failing to replicate is 0.5. It is worthwhile to clarify two points about this probability ratio. First, we assume that the probability of failing to replicate is less if the original finding is true than if it is not true, so that the ratio ought to be substantially less than 1. Second, how much less than 1 this ratio will be depends largely on the quality of the replication; as the replication becomes closer to meeting the ideal *ceteris paribus* condition, the ratio will deviate increasingly from 1. Put more generally, as the quality of the auxiliary assumptions going into the replication attempt increases, the ratio will decrease. Given these two ratios of 50 and 0.5, the posterior confidence ratio is 25. Although this is a substantial decrease in confidence from 50, the aficionado still believes that the finding is extremely likely to be true. But suppose there is another replication failure and the probability ratio is 0.8. In that case, the new confidence ratio is (25)(0.8) = 20. The pattern should be clear here: As there are more replication failures, a rational person, even if that person is an aficionado of the original researcher, will experience continually decreasing confidence as the replication failures mount.

If we imagine that there are *N* attempts to replicate the original finding that fail, the process described in the foregoing paragraph can be summarized in a single equation that gives the ratio of posterior confidences in the original finding, given that there have been *N* failures to replicate. This is a function of the prior confidence ratio and the probability ratios in the first replication failure, the second replication failure, and so on.

(3) $$\begin{array}{l} \frac{p(T|F_{N})}{p(\sim T|F_{N})}=\frac{p(T)}{p(\sim T)}\frac{p(F_{1}|T)}{p(F_{1}|\sim T)}\frac{p(F_{2}|T)}{p(F_{2}|\sim T)}\ldots \\ \frac{p(F_{N}|T)}{p(F_{N}|\sim T)}=\frac{p(T)}{p(\sim T)}\prod_{i\;=\;1}^{N}\frac{p(F_{i}|T)}{p(F_{i}|\sim T)} \end{array}$$

For example, staying with our aficionado with a prior confidence ratio of 50, imagine a set of 10 replication failures, with the following probability ratios: 0.5, 0.8, 0.7, 0.65, 0.75, 0.56, 0.69, 0.54, 0.73, and 0.52. The final confidence ratio, according to Equation (3), would be:

$$\begin{array}{l} \left(50)(0.5)(0.8)(0.7)(0.65)(0.75)(0.56)(0.69)(0.54)(0.73)(0.52\right)\\ \;=0.54. \end{array}$$

Note the following. First, even with an extreme prior confidence ratio (we had set it at 50 for the aficionado), it is possible to overcome it with a reasonable number of replication failures providing that the person tallying the replication failures is a rational Bayesian (and there is reason to think that those attempting the replications are sufficiently competent in the subject area and methods to be qualified to undertake them). Second, it is possible to go from a state of extreme confidence to one of substantial lack of confidence. To see this in the example, take the reciprocal of the final confidence ratio (0.54), which equals 1.84. In other words, the Bayesian aficionado now believes that the finding is 1.84 times as likely to be not true as true. If we imagine yet more failed attempts to replicate, it is easy to foresee that the future belief that the original finding is not true could eventually become as powerful, or more powerful, than the prior belief that the original finding is true.

In summary, auxiliary assumptions play a role, not only for original theory-testing experiments but also in replications—even in replications concerned only with the original finding and not with the underlying theory. A particularly important auxiliary assumption is the ever-present *ceteris paribus* assumption, and the extent to which it applies influences the “convincingness” of the replication attempt. Thus, a change in confidence in the original finding is influenced both by the quality and quantity of the replication attempts, as Equation (3) illustrates.

In presenting Equations (1–3), we reduced the theoretical content as much as possible, and more than is realistic in actual research^10^, in considering so-called “direct” replications. As the replications serve other purposes, such as “conceptual” replications, the amount of theoretical content is likely to increase. To link that theoretical content to the replication attempt, more auxiliary assumptions will become necessary. For example, in a conceptual replication of an experiment finding that attitudes influence behavior, the researcher might use a different attitude manipulation or a different behavior measure. How do we know that the different manipulation and measure are sufficiently theoretically unimportant that the conceptual replication really is a replication (i.e., a test of the underlying theory)? We need new auxiliary assumptions linking the new manipulation and measure to the corresponding constructs in the theory, just as an original set of auxiliary assumptions was necessary in the original experiment to link the original manipulation and measure to the corresponding constructs in the theory. Auxiliary assumptions always matter—and they should be made explicit so far as possible. In this way, it will be easier to identify where in the chain of assumptions a “breakdown” must have occurred, in attempting to explain an apparent failure to replicate.

## Conclusion

Replication is not a silver bullet. Even carefully-designed replications, carried out in good faith by expert investigators, will never be conclusive on their own. But as Tsang and Kwan (1999) point out:

If replication is interpreted in a strict sense, [conclusive] replications or experiments are also impossible in the natural sciences.… So, even in the “hardest” science (i.e., physics) complete closure is not possible. The best we can do is control for conditions that are plausibly regarded to be relevant. (p. 763)

Nevertheless, “failed” replications, especially, might be dismissed by an original investigator as being flawed or “incompetently” performed—but this sort of accusation is just too easy. The original investigator should be able to describe exactly what parameters she sees as being theoretically relevant, and under what conditions her “effect” should obtain. If a series of replications is carried out, independently by different labs, and deliberately tailored to the parameters and conditions so described—yet they reliably fail to produce the original result—then this should be considered *informative*. At the very least, it will suggest that the effect is sensitive to theoretically-unspecified factors, whose specification is sorely needed. At most, it should throw the existence of the effect into doubt, possibly justifying a shift in research priorities. Thus, while “falsification” can in principle be avoided ad infinitum, with enough creative effort by one who wished to defend a favored theory, scientists should not seek to “rescue” a given finding at any empirical cost^11^. Informative replications can reasonably factor into scientists' assessment about just what that cost might be; and they should pursue such replications as if the credibility of their field depended on it. In the case of experimental social psychology, it does.

### Conflict of interest statement

The authors declare that the research was conducted in the absence of any commercial or financial relationships that could be construed as a potential conflict of interest.

## Footnotes

^1^There are two steps to understanding this idea. First, because the foundational theories are so insecure, and the field's findings so under dispute, the “correct” empirical outcome of a given experimental design is unlikely to have been firmly established. Second, and insofar the first step applies, the standard by which to judge whether a replication has been competently performed is equally unavailable—since that would depend upon knowing the “correct” outcome of just such an experiment. Thus, a “competently performed” experiment is one that produces the “correct” outcome; while the “correct” outcome is defined by whatever it is that is produced by a “competently performed” experiment. As Collins (1985) states: “Where there is disagreement about what counts as a competently performed experiment, the ensuing debate is coextensive with the debate about what the proper outcome of the experiment is” (p. 89). This is the infamously circular *experimenter's regress*. Of course, a competently performed experiment should produce satisfactory (i.e., meaningful, useful) results on “outcome neutral” tests.

^2^Assuming that it is a psychology experiment. Note that even if the “same” participants are run through the experiment one more time, they'll have changed in at least one essential way: they'll have already gone through the experiment (opening the door for practice effects, etc.).

^3^On Popper's view, one must set up a “falsifying hypothesis,” i.e., a hypothesis specifying how another experimenter could recreate the falsifying evidence. But then, Popper says, the falsifying hypothesis itself should be severely tested and corroborated before it is accepted as falsifying the main theory. Interestingly, as a reviewer has suggested, the distinction between a falsifying hypothesis and the main theory may also correspond to the distinction between direct vs. conceptual replications that we discuss in a later section. On this view, direct replications (attempt to) reproduce what the falsifying hypothesis states is necessary to generate the original predicted effect, whereas conceptual replications are attempts to test the main theory.

^4^The percentages reported here are the geometric mean of self-admission rates, prevalence estimates by the psychologists surveyed, and prevalence estimates derived by John et al. from the other two figures.

^5^Priming has been defined a number of different ways. Typically, it refers to the ability of subtle cues in the environment to affect an individual's thoughts and behavior, often outside of her awareness or control (e.g., Bargh and Chartrand, 1999).

^6^Even more damning, Trafimow (2003; Trafimow and Rice, 2009; Trafimow and Marks, 2015) has argued that the standard significance tests used in psychology are invalid even when they are done “correctly.” Thus, even if psychologists were to follow the prescriptions of Simmons et al.—and reduce their researcher degrees of freedom (see the discussion following this footnote)—this would still fail to address the core problem that such tests should not be used in the first place.

^7^For example, a “study found that eating disorder patients were significantly more likely than others to see frogs in a Rorschach test, which the author interpreted as showing unconscious fear of oral impregnation and anal birth…” (Giner-Sorolla, 2012, p. 562).

^8^Asendorpf et al. (2013) explain why this is so: “[direct] replicability is a *necessary condition* for further generalization and thus indispensible for building solid starting points for theoretical development. Without such starting points, research may become lost in endless fluctuation between alternative generalization studies that add numerous boundary conditions but fail to advance theory about why these boundary conditions exist” (p. 140, emphasis added).

^9^There may be other reasons why the “failed” replication by Doyen et al. should not be considered conclusive, of course; for further discussion see, e.g., Lieberman (2012).

^10^Indeed, we have presented our analysis in this section in abstract terms so that the underlying reasoning could be seen most clearly. However, this necessarily raises the question of how to go about implementing these ideas in practice. As a reviewer points out, to calculate probabilities, the theory being tested would need to be represented as a probability model; then in effect one would have Bayes factors to deal with. We note that both Dienes (2014) and Verhagen and Wagenmakers (2014) have presented methods for assessing the strength of evidence of a replication attempt (i.e., in confirming the original result) along these lines, and we refer the reader to their papers for further consideration.

^11^As Doyen et al. (2014, p. 28, internal references omitted) recently argued: “Given the existence of publication bias and the prevalence of questionable research practices, we know that the published literature likely contains some false positive results. Direct replication is the only way to correct such errors. The failure to find an effect with a well-powered direct replication must be taken as evidence against the original effect. Of course, one failed direct replication does not mean the effect is non-existent—science depends on the accumulation of evidence. But, treating direct replication as irrelevant makes it impossible to correct Type 1 errors in the published literature.”
